# DMFT of the First Permanent Molars, dmft and Related Factors among All First-Grade Primary School Students in Rafsanjan Urban Area

**DOI:** 10.30476/DENTJODS.2020.85573.1136

**Published:** 2021-06

**Authors:** Nazanin Kamiab, Yasaman Mohammadi Kamalabadi, Mahmood Sheikh Fathollahi

**Affiliations:** 1 Dept. of Oral Medicine, School of Dentistry, Rafsanjan University of Medical sciences, Rafsanjan, Iran; 2 General Dentist; School of Dentistry, Rafsanjan University of Medical sciences, Rafsanjan, Iran; 3 Dept. of Biostatistics, Rajaie Cardiovascular Medical and research center, Iran University of Medical Science, Tehran, Iran

**Keywords:** Dental caries, Family size, Gender, Oral hygiene, Primary school

## Abstract

**Statement of the Problem:**

Dental caries is the most common chronic childhood disorders throughout the world. The dmft (decayed, missing, and filled primary teeth) and DMFT (decayed, missing, and filled permanent teeth) are some of the most important epidemiological indices in dentistry. Evaluation of these two indicators in the population can help in future planning of healthcare programs to improve oral health status.

**Purpose:**

The aim of this study was to evaluate these indicators and the related factors in first-grade primary school students in Rafsanjan urban area to determine their present status, which might be helpful for future health care planning.

**Materials and Method:**

In this cross-sectional study, DMFT index of first permanent molar and dmft were evaluated by census method on 2031 first-grade primary school students in Rafsanjan urban area in 2018 (May-June). Dental examination was done using a mirror and probe under natural light according to World Health Organization criteria. The data were then analyzed using independent two-sample t-test, one-way ANOVA, Tukey's multiple comparisons test, Kolmogorov-Smirnov nonparametric test and Leven's test in SPSS version 21 software.

**Results:**

The mean and standard deviation of dmft index and DMFT index of first permanent molar were 6.37 ± 3.40 and 0.30 ± 0.72, respectively. The proportion of caries free students was 4.1%. A significant association was found between the values of these indices and school type, the level of education of parents, parental occupation, family size, and frequency of brushing and the use of floss (*p* < 0.05). However, there was no significant association between these two indices with gender (*p*= 0.347 and *p*= 0.593, respectively).

**Conclusion:**

The results of this study showed high prevalence of caries in first-grade primary school students in Rafsanjan. Therefore, to improve this situation, more attention is needed for proper oral health program planning and education of families concerning oral hygiene and dental preventive measures.

## Introduction

Dental caries is the most common preventable chronic childhood disease worldwide, which is an infectious and multifactorial disease [ 1]. Primary teeth play an important role in meeting the nutritional and developmental needs of children. Moreover, dental caries can pose negative effects on children's growth, health status, quality of life, speech and communication and their ability to eat. Therefore, prevention of dental caries in children is very essential [ 2- 4]. The results of one study showed that 298 billion dollars annually is spent on direct dental costs worldwide, accounting for 4.6% of total health spending [ 5]. The prevalence and severity of dental caries in children worldwide has decreased, especially in developed countries [ 6], while the prevalence of dental caries in developing countries is increasing [ 7]. The causes of dental caries may be due to poor socioeconomic status, cultural habits, failure to prevent oral disease, lack of fluoride in water, use of sugar products, and poor oral hygiene [ 8- 9].

The DMFT index of permanent teeth and the dmft index for primary teeth that include decayed (D/d), missing (M/m) and filled (F/f) teeth are important indicators in assessing the health of children in each community [ 10]. The dmft index in Iran is high compared to the World Health Organization standards [ 11]. Moreover, according to the report of the Deputy of Health, caries index in Iran in the age group of 6 years has increased from 5 in 2006 to 5.7 in 2011 [ 12]. This age group refers to the dentist less frequently than others do for regular periodic examinations. Caries also affects primary teeth far more than permanent teeth, which can be attributed to differences in enamel structure, oral hygiene and less preventive measures. In addition, having caries early in life is a predictor of the risk of caries in adulthood [ 13- 15].

Numerous factors have been shown to be related to dmft index such as gender, tooth brushing, parents’ occupation, level of education, and family size [ 16- 19]. The study of Emamian *et al*. [ 18], which was conducted on 5620 6- to 12-year-old students, indicated dental status of girls was poorer compared to boys. A study in Yasuj showed that the higher the socioeconomic status of the families, the lesser the dmft and DMFT indices in children [ 19]. Frequent toothbrushing and dental flossing was shown to have a positive effect on dental health in studies by Ramezani *et al*. [ 20] and Abdelhamid *et al*. [ 21]. They reported that students who brushed their teeth twice a day or more and those who flossed (whose number was relatively low) had lower DMFT score in comparison with students who did no brush their teeth regularly and did not use dental floss [ 20- 21].

Despite advances in science and technology, none of the introduced materials in dentistry is as ideal as normal tooth tissue. Thus, prevention is the best way to address the issue of tooth decay [ 22]. To take oral disease preventive measures in each area, it is essential that the oral health status of that area be determined initially. Surveying children in schools is the most common way of collecting information in societies [ 23]. Since the mean dmft index changes over time and it is needed to have access to new information for future planning, the evaluation of this index should be repeated. Hence, in this study, the index was measured in all first-grade primary school students of Rafsanjan urban area for the first time. The aim of this study, which is conducted to pave the way for future prevention programs, was to determine DMFT index of first permanent molars and dmft and the associated factors in all first-grade primary school students in urban area of Rafsanjan in 2018.

## Materials and Method

In this cross-sectional study, out of a total of 2105 first-grade primary school students of Rafsanjan urban area, 2031 students were examined by census method after being approved in the Ethics Committee of Rafsanjan University of Medical Sciences (code: IR.RUMS.REC. 1398.064) and in collaboration with Rafsanjan primary schools and education management in 2018 (May-June).

Accordingly, the overall goals of the project were communicated to the schools, and parents were informed of these goals during a meeting held by the school health educator. After obtaining written consent from them, the appropriate time for the examination was determined in collaboration with schools’ principals.

Inclusion criteria included six to seven years of age, school attendance on examination day, child cooperation and parental consent, and exclusion criteria included children with enamel and dentin aplasia, amelogenesis imperfecta, dentinogenesis imperfecta, other dental genetic abnormalities, diseases and drugs that reduce salivary secretion (antihypertesives, antihistamines, antidepressants, antipsychotics, antiemetics, antispasmotics and anti-parkinsonian drugs) and the presence of orthodontic appliance [ 24- 25].

The questionnaire used in this study consisted of three sections including 1) examination date and school name, 2) demographic and oral hygiene information (name, gender and date of birth, number of family members, birth rank, education and occupation of parents, use of dental floss and frequency of toothbrushing per day), and 3) dental status (in form of a chart). The reliability and validity of this questionnaire were confirmed in previous studies [ 26- 27]. 

In order to answer the questions related to students’ age, number of family members, birth rank and occupation and education of parents, the students' health profile was used and the students themselves were asked about behavioral questions (brushing and flossing); however, teachers confirmed the accuracy of the answers based on information they had obtained from parents.

Examination of teeth was done under natural light using a disposable dental mirror (Atlas, Tehran, Iran) and community periodontal index (CPI) probe (HU Friedy, Chicago, USA) and when necessary, flashlight and explorer were used. The criteria for decay, filling and loss of teeth were based on the standards defined by the World Health Organization so that teeth with damaged surfaces or pit and grooves, cavitated enamel and softened surfaces (felt by the probe) were considered as decayed. Any tooth that was dressed with one of the temporary fillings and any tooth, which was filled but had caries, was considered decayed. White spots were not considered as caries. Teeth that were not present only due to caries were considered missing and teeth that were not present due to orthodontics, accident, and so on were not included. A tooth with one or more surfaces that had permanent filling and no old or new caries was considered as filled. The first permanent molar tooth with intact sealant was considered healthy. Teeth with faulty sealants were considered decayed [ 12].

All examinations were performed by a dental student and another dental student was responsible for recording information and completing the questionnaire. The dental students received the necessary training in examinations and calculations prior to the examination under the supervision of a pediatric dentist and their reliability was confirmed before the main study. Data were categorized and coded for statistical analysis in SPSS version 21.

### Statistical analysis method

Data were analyzed using SPSS software (version 21). Results were reported as "mean± standard deviation (SD)" for quantitative variables and as "number (percentage)" for qualitative variables.

Kolmogorov-Smirnov nonparametric test showed that frequency distribution of DMFT index of first permanent molars and dmft and their components met normal distribution (*p*> 0.05). Leven's test for homogeneity of variances also indicated that the variance of DMFT index of first permanent molars and dmfts and their components across the studied groups (number of households, parental education, parental occupation and frequency of brushing days) did not significantly differ (*p*> 0.05). Significance level was set at 0.05.

Independent two-sample t-test was used to compare the mean DMFT index of first permanent molars and dmft and their components across students according to school type, gender, mother's occupation, and dental flossing. One-way ANOVA was applied to compare the mean DMFT index of first permanent molars and dmft and their components in students by number of family members, birth rank, parents’ educational level, father's occupation and frequency of toothbrushing per day. Tukey's multiple comparisons test was performed when a significance result took place in the one-way ANOVA test.

## Results

In this study, 2031 first-grade primary school students of Rafsanjan were studied. A total of 1836 (90.4%) of the students had decayed primary teeth, 955 (47.1%) of them had missed primary teeth, and 564 (27.8%) of them had filled primary teeth. A total of 364 (17.9%) of the students had decayed first permanent molars. Four (0.2%) and 18 (0.8%) students had permanent missed (M) and permanent filled (F) teeth, respectively. The mean of these two components was not included in the statistical analysis because of their low value. In examination of first permanent molars out of 2031 students, 11 (0.5%) had none of the first permanent molars and 1619 (79.7%) had four sound first permanent molars. Also 83 (4.1%) of students were caries free.

The minimum amount of all parameters was zero and the maximum amounts of d, m, f, dmft, D and DMFT index of first permanent molars were 16,16, 8, 20, 4 and 4, respectively. The mean±SD for these indices were 4.49±3.07, 1.16±1.77, 0.72±1.45, 6.37±3.40, 0.29±0.70 and 0.30±0.72, respectively.

According to Table 1, the independent two-sample t-test showed that the means of d, f, and dmft components in students of public schools were significantly higher than students of private schools (*p*< 0.05), while the mean of component f in students of private schools was significantly higher than students in public schools (*p*= 0.006). The results also showed that the means of other components were not significantly different across the school type (*p*> 0.05).

**Table1:** Comparison of mean dmft and DMFT index of first permanent molars and their components in first-grade primary school students of public and private schools of Rafsanjan city in 2018 (n = 2031)

School type	Public school (n=1613) Mean ± Standard Deviation	Private school (n=418) Mean ± Standard Deviation	*p* Value
Index			
d	4.59±3.05	4.13±2.94	0.006
m	1.19±1.76	1.03±1.82	0.109
f	0.67±1.40	0.91±1.59	0.006
dmft	6.45±3.42	6.07±3.33	0.045
D6	0.29±0.72	0.28±0.65	0.803
DMFT6	0.30±0.73	0.29±0.67	0.797

d: decayed, m: missing, f: filled (for primary teeth)

dmft: decayed, missing and filled teeth (for primary teeth)

D6: decayed first permanent molar

DMFT6: decayed, missing and filled first permanent molar

As shown in Table 2, independent two-sample t- test showed that the mean of missing primary teeth in male students was significantly higher than female students (*p*= 0.036). The means of other components in the two genders showed no significant difference (*p*< 0.05). The average number of family members was 4.56±1.43 ranged 3 to 14 people. One-way ANOVA illustrated that the means of all components in students with different number of family members were statistically significant (*p*< 0.05), so that with increasing family size, the mean of d, m, dmft, D and DMFT index of first permanent molars increased significantly (*p*< 0.05), while the mean of component f significantly decreased (*p*< 0.001). In addition, one-way ANOVA showed that the means of d, f and dmft in students with different birth ranks were significantly different (*p*< 0.05), so that with birth rank increase the means of d and dmft increased, while the mean of component f decreases significantly. The means of other components were not statistically different (*p*> 0.05).

**Table2:** Comparison of mean dmft and DMFT index of first permanent molars and their components according to gender, number of family members and birth order in first-grade primary school students in Rafsanjan city in 2018 (n = 2031)

Variable	Index	d	m	f	dmft	D6	DMFT6
Gender	Female (n=934) Mean ± SD	4.49±3.14	1.07±1.64	0.73±1.46	6.29±3.45	0.30±0.74	0.31±0.75
	Male (n=1097) Mean ± SD	4.49±2.93	1.23±1.87	0.71±1.44	6.43±3.36	0.28±0.67	0.29±0.69
*p* Value		0.967	0.036	0.674	0.347	0.550	0.593
Number of family members	Three (n=272) Mean ± SD	4.08±3.02	1.01±1.74	0.94±1.60	6.03±3.40	0.23±0.63	0.24±0.64
	Four (n=1037) Mean ± SD	4.33±3.03	1.09±1.75	0.91±1.61	6.33±3.43	0.25±0.66	0.26±0.68
	Five (n=424) Mean ± SD	4.57±3.07	1.19±1.66	0.50±1.13	6.85±3.31	0.34±0.75	0.36±0.77
	Six (n=133) Mean ± SD	4.80±2.75	1.51±2.05	0.23±0.92	6.95±3.34	0.39±0.75	0.39±0.75
	>six (n=165) Mean ± SD	5.22±2.89	1.67±1.93	0.08±0.55	7.18±3.37	0.49±0.85	0.41±0.88
*p* Value		<0.001	0.006	<0.001	0.002	0.008	0.008
Birth rank	Single child (n=271) Mean ± SD	4.08±3.02	1.01±1.75	0.94±1.61	6.03±3.41	0.23±0.63	0.24±0.64
	First child (n=676) Mean ± SD	4.22±2.91	1.11±1.82	0.87±1.59	6.20±3.28	0.30±0.71	0.32±0.72
	Middle child (n=354) Mean ± SD	4.79±2.97	1.30±1.89	0.72±1.42	6.34±3.45	0.36±0.76	0.37±0.78
	Last child (n=730) Mean ± SD	4.97±3.13	1.19±1.67	0.25±0.87	6.67±3.47	0.27±0.69	0.28±0.71
*p* Value		<0.001	0.178	<0.001	0.017	0.118	0.117

d: decayed, m: missing, f: filled (for primary teeth)

dmft: decayed, missing and filled teeth (for primary teeth)

D6: decayed first permanent molar

DMFT6: decayed, missing and filled first permanent molar

SD: standard deviation

According to Table 3, one-way ANOVA revealed that the means of all components in first-grade primary school students were statistically significant (*p*< 0.001).

**Table3:** Comparison of mean dmft and DMFT index of first permanent molars and their components according to parent’s level of education and occupation in first-grade primary school students in Rafsanjan city in 2018 (n = 2031)

Variable	Index	d	m	f	dmft	D6	DMFT6
Father’s level of education	Illiterate + primary education (n=255) Mean±SD	5.41±3.24	1.56±2.05	0.24±0.90	7.20±3.48	0.57±0.95	0.58±0.97
	Secondary education (n=309) Mean ± SD	6.03±3.06	1.71±2.03	0.47±1.29	8.21±3.05	0.46±0.85	0.47±0.86
	Higher secondary education (n=800) Mean ± SD	4.51±2.78	1.06±1.66	0.71±1.39	6.28±3.13	0.24±0.61	0.25±0.64
	College education (n=649) Mean ± SD	3.39±2.79	0.85±1.54	1.04±1.67	5.28±3.43	0.16±0.54	0.17±0.56
*p* Value		<0.001	<0.001	<0.001	<0.001	<0.001	<0.001
Mother’s level of education	Illiterate + primary education (n=259) Mean ± SD	5.43±3.11	1.56±1.90	0.19±0.67	7.18±3.34	0.57±0.99	0.58±1.01
	Secondary education (n=308) Mean ± SD	5.79±3.10	1.75±2.20	0.60±1.41	8.13±3.27	0.46±0.80	0.47±0.81
	Higher secondary education (n=1049) Mean ± SD	4.51±2.87	1.07±1.60	0.77±1.47	6.35±3.18	0.23±0.63	0.2±0.65
	College education (n=411) Mean ± SD	2.88±2.55	0.67±1.52	1.20±1.66	4.57±3.21	0.13±0.46	0.15±0.50
*p* Value		<0.001	<0.001	<0.001	<0.001	<0.001	<0.001
Father’s occupation	Manual worker (n=175) Mean ± SD	4.78±3.06	1.47±1.99	0.19±0.84	6.57±3.37	0.47±0.91	0.50±0.93
	Employee (n=479) Mean ± SD	3.88±2.93	1.01±1.70	1.14±1.72	6.02±3.47	0.21±0.60	0.22±0.61
	Self-employed (n=1285) Mean ± SD	4.49±2.83	1.18±1.77	0.61±1.32	6.14±3.28	0.29±0.70	0.31±0.72
	Others (n=74) Mean ± SD	3.59±3.06	0.85±1.39	1.30±1.93	5.47±3.76	0.19±0.61	0.23±0.67
*p* Value		<0.001	0.010	<0.001	0.005	<0.001	<0.001
Mother’s occupation	Housewife (n=1624) Mean ± SD	4.58±3.4	1.20±1.80	0.66±1.39	6.54±3.43	0.31±0.73	0.32±0.74
	Employed (n=403) Mean ± SD	4.13±2.94	0.96±1.61	0.98±1.65	6.07±3.27	0.19±0.58	0.22±0.62
*p* Value		0.008	0.010	<0.001	0.047	0.001	0.006

d: decayed, m: missing, f: filled (for primary teeth)

dmft: decayed, missing and filled teeth (for primary teeth)

D6: decayed first permanent molar

DMFT6: decayed, missing and filled first permanent molar

SD: standard deviation

The means of d, m, dmft, D and DMFT index of first permanent molar teeth decreased significantly with increasing level of parents’ education (*p*< 0.001), while the mean of component f increased significantly (*p*< 0.001). Furthermore, one-way ANOVA showed that the means of d, m, dmft, D and DMFT index of first permanent molars were the highest in students whose fathers were manual workers and the lowest in students whose fathers had other jobs (*p*< 0.05). Moreover, the mean of component f was the lowest in students whose fathers were manual workers and the highest in students whose fathers had other jobs (*p*< 0.001). The independent two-sample t-test suggested that the means of all components were significantly different in terms of mothers’ occupation (*p*< 0.05). The mean of d, m, dmft, D and DMFT of first permanent molars in students whose mothers were housewives were significantly higher than those of students whose mothers were employed (*p*< 0.05), while the mean of component f in students with employed mothers was significantly higher than those of students whose mothers were housewives (*p*< 0.001).

The mean of the frequency of toothbrushing per day was 0.69±0.70 and varied from zero to three times a day. Out of all studied subjects, 109 (5.4%) of the students studied used dental floss and 1922 (94.6%) did not. According to Table 4, one-way ANOVA for brushing and independent two-sample t-test for using dental floss showed that the mean of all components in students who brushed their teeth more frequently and those who used dental floss were significantly less (*p*< 0.001).

**Table4:** Comparison of mean dmft and DMFT index of first permanent molars and their components according to oral hygiene practices in first-grade primary school students in Rafsanjan city in 2018 (n = 2031)

Oral hygiene practices	Index	d	m	f	dmft	D6	DMFT6
frequency of toothbrushingper day	Zero (n=890) Mean ± SD	6.38±2.85	1.86±2.14	0.83±1.50	8.93±2.50	0.46±0.86	0.48±0.87
	One (n=905) Mean ± SD	3.45±2.20	0.73±1.25	0.68±1.45	5.01±2.37	0.18±0.57	0.19±0.59
	More than one (n=236) Mean ± SD	1.37±1.56	0.14±0.44	0.46±1.18	1.97±1.81	0.04±0.19	0.05±0.23
Flossing	Yes (n=109) Mean ± SD	1.21±1.53	0.17±0.46	0.27±0.37	2.10±1.91	0.01±0.09	0.03±0.21
	No (n=1922) Mean ± SD	4.68±2.99	1.21±1.80	0.72±1.45	6.61±3.31	0.30±0.72	0.32±0.74
*p* Value		<0.001	<0.001	<0.001	<0.001	<0.001	<0.001

d: decayed, m: missing, f: filled (for primary teeth)

dmft: decayed, missing and filled teeth (for primary teeth)

D6: decayed first permanent molar

DMFT6: decayed, missing and filled first permanent molar

SD: standard deviation

## Discussion

Dental caries is a transmissible infectious disease that begins with the activity of bacteria on the surface of the tooth. The most common epidemiological scale for determining dental status is DMFT index of permanent teeth and dmft for primary teeth [ 10]. It is necessary to evaluate dmft and DMFT in different age groups from time to time and in different regions to check the trend of changes in these indices and evaluate the measures taken in the country. Periodic studies are also needed to assess the status of oral health and to provide statistics and supporting documents for the preparation and implementation of the necessary health, care, and treatment programs. Thus, it was decided to evaluate the dmft index and DMFT index of first permanent molars in first-grade primary school students in Rafsanjan.

Based on the results of this study, the mean dmft and DMFT index of first permanent molars were 6.37 and 0.30 in first-grade primary school students of Rafsanjan city, respectively. The mean dmft index in public schools was significantly greater than private schools. However, the number of primary filled teeth was significantly higher in private schools, which may be due to the higher socio-economic level of parents of students in private schools. In general, it can be stated that the role of schools and families is very important in thinking about special measures to improve the oral health of children. Especially in this group of students who have sufficient physical and mental abilities, parental attention is also an additional help in this regard. This result was in line with the result reported by Priyanka *et al*. [ 28] and in contrast to the result of Sofola *et al*. [ 29]. This discrepancy can be due to differences in socio-cultural levels as well as access to caries prevention services, including systemic fluoride in different cities, which reduces the gap between public and private schools.

In the current study, no statistically significant difference was found between dmft and DMFT index of first permanent molars according to gender, which was comparable to the results of Đuričković *et al*. [ 30]. This result may be due to the greater importance of personal hygiene than gender differences in caries incidence. These results are inconsistent with the study of Wang *et al*. [ 31], which can be attributed to the cultural differences in the communities concerning different interest for oral health care in different genders.

According to the results, the mean dmft, D for first permanent molars and DMFT index of first permanent molars increased significantly with increasing number of family members, which is consistent with the study of Javadinejad *et al*. [ 32]. Christensen *et al*. [ 33] also found that in families with fewer children, caries was less common. In fact, with the smaller number of children, parents have more opportunity to take care of their children's oral health and perform better in regular visits to the dentist. The increase in the number of children in a family may also affect the economic situation and decrease the attention to children concerning the lack of time and facilities in large families.

The present study showed an inverse relationship between birth rank and dmft index. Children who were the first child of the family had significantly less caries than those who were the second, third, and so on. Parents seem to be more concerned in taking care of their first child's oral health. This finding was similar to the study of Sajadi *et al*. [ 34] but Namal *et al*. [ 35] showed the opposite. With the increase in the number of children, the number of filled teeth was significantly reduced, which could be due to the limitation in the economic status of parents and time devoted to each child for dental cares as the number of family members increased.

With increasing parents’ level of education, the mean dmft and DMFT index of first permanent molar were significantly reduced. This finding was similar to the study by Stephen *et al*. [ 36]; they found that there was a significant relationship between father's education level and the low risk of caries in children. Ismail and Sohn [ 37] also found that children whose parents had a college education had significantly lower caries than children whose parents had lower levels of education. The rationale behind it could be that higher educational level broadens parents’ knowledge of oral health. These results contradict the study of Auad *et al*. [ 38]. The reason for this conflict can be ascribed to the increased likelihood of mothers being employed with higher education levels and thus less time and attention paid to children's oral health care in this study.

The means of dmft and DMFT index of first permanent molars were significantly higher in children whose fathers were self-employed and manual workers. In the study of Ghandehari Motlagh *et al*. [ 39] father’s job had no significant effect on child’s dmft, although the highest dmft was found in children whose fathers were manual workers and the lowest dmft was in children whose fathers were employees. Yousofi *et al*. [ 19] reported results similar to our study. This goes back to the level of awareness and then the economic problems of families because manual workers generally have less education and less awareness as well as more problems and deprivations that will certainly affect their children's oral health status.

The dmft and DMFT index of first permanent molars were significantly more frequent in students whose mothers were housewives than in students with employed mothers. In the study of Ghandehari Motlagh *et al*. [ 39], the highest mean dmft was found in children whose mothers were housewives (3.21) and the mean dmft in children whose mothers were employed was 1.92. The findings indicate that employed mothers benefit from more social education and that they are generally more educated than housewives, which can result in better dental status of their children. This result contradicts the study of Nematollahi *et al*. [ 40] who claimed that employed mothers do not care for their children’s oral health because of their time constraints.

The mean dmft and DMFT index of the first permanent molar teeth were significantly decreased with increasing brushing frequency per day. There was also a significant relationship between the frequency of brushing and dmft in the study of Abdelhamid *et al*. [ 21] and Faezi *et al*. [ 25] confirming the results of this study. The result was inconsistent with the study by Yousofi *et al*. [ 19], which may be due to insufficient skill, and training of students in brushing and microbial plaque removal.

In this study, children who used dental floss had significantly lower dmft and DMFT index of first permanent molars than children who did not use dental floss. This indicates the importance of flossing in reducing dental caries, especially in posterior teeth. This result is consistent with the study of Ramezani *et al*. [ 20]. In the study of Yousofi *et al*. [ 19], there was no significant difference between dmft in terms of dental floss usage. Since the positive effect of dental flossing is obtained when the dental floss is applied correctly, this difference may be due to differences in the use of dental floss in the two studies.

Limitations of this study include the lack of investigation of the relationship between nutrition, regular dental check-up, and fluoride supplementation with dental caries. Strengths of our study include the large sample size, in which all first-grade primary students of urban area of Rafsanjan were studied, and also a trained dental student examined teeth and the data were not based on self or parents’ report. Further studies are needed to evaluate dental status of students at the age of 12. Moreover, investigation of other effective factors on dental caries seems crucial.

## Conclusion

The results of this study showed that the mean dmft and DMFT index of first permanent molar teeth in first-grade primary school students of Rafsanjan were almost similar to previous studies in this area. There was a significant relationship between the number of family members, parents’ level of education, parents’ occupation, and type of school, frequency of toothbrushing and use of dental floss with dental caries. No significant relationship was found between gender and caries. It seems necessary to implement an oral health prevention program for students in this zone.
